# Risk of Second Tumors in Retinoblastoma Survivors after Ionizing Radiation: A Review

**DOI:** 10.3390/cancers15225336

**Published:** 2023-11-09

**Authors:** Diana Figueiredo, Inês A. Marques, Ana Salomé Pires, Claudia F. Cavaleiro, Luís C. Costa, Guilherme Castela, Joaquim N. Murta, Maria Filomena Botelho, Ana Margarida Abrantes

**Affiliations:** 1 University of Coimbra, Coimbra Institute for Clinical and Biomedical Research (iCBR) Area of Environment Genetics and Oncobiology (CIMAGO), Institute of Biophysics, Faculty of Medicine, 3000-548 Coimbra, Portugal; fgrd.diana@gmail.com (D.F.); iafmarques@uc.pt (I.A.M.); pireslourenco@uc.pt (A.S.P.); 2 University of Coimbra, Faculty of Sciences and Technology, 3000-548 Coimbra, Portugal; 3 University of Coimbra, Center for Innovative Biomedicine and Biotechnology (CIBB), 3000-548 Coimbra, Portugal; 4 University of Coimbra, Faculty of Pharmacy, 3000-548 Coimbra, Portugal; 5 Clinical Academic Centre of Coimbra (CACC), 3000-061 Coimbra, Portugal; 12583@chuc.min-saude.pt (G.C.); jmurta@fmed.uc.pt (J.N.M.); 6 Medical Imaging and Radiotherapy Department, Polytechnic Institute of Coimbra, ESTESC-Coimbra Health School, 3045-093 Coimbra, Portugal; claudiacavaleiro@estescoimbra.pt (C.F.C.); luiscosta@estescoimbra.pt (L.C.C.); 7 Pediatric Oncology Service, Centro Hospitalar Universitário de Coimbra, 3000-602 Coimbra, Portugal; 8 Department of Ophthalmology, Centro de Responsabilidade Integrado de Oftalmologia, Centro Hospitalar e Universitário de Coimbra, 3000-602 Coimbra, Portugal; 9 University of Coimbra, Faculty of Medicine, 3000-548 Coimbra, Portugal

**Keywords:** retinoblastoma, *RB1* gene, ionizing radiation, second malignant neoplasms

## Abstract

**Simple Summary:**

Retinoblastoma is the most common ocular tumor among children. Typically, individuals with retinoblastoma are regularly monitored with diagnostic techniques and treated with many therapeutic approaches that use ionizing radiation. Thus, it is extremely important to clarify the effects of ionizing radiation on these patients, since they seem to be at a higher risk to develop other types of tumors compared to other cancer patients, especially when treated with radiation therapy. Many studies have been performed with the aim of elucidating the relationship between ionizing radiation exposure and the incidence of other malignant tumors. The results seem to show no significant correlation.

**Abstract:**

Retinoblastoma (RB) is the most common ocular neoplasm in children, whose development depends on two mutational events that occur in both alleles of the retinoblastoma susceptibility gene (*RB1*). Regarding the nature of these mutational events, RB can be classified as hereditary if the first event is a germline mutation and the second one is a somatic mutation in retina cells or nonhereditary if both mutational events occur in somatic cells. Although the rate of survival of RB is significantly elevated, the incidence of second malignant neoplasms (SMNs) is a concern, since SMNs are the main cause of death in these patients. Effectively, RB patients present a higher risk of SMN incidence compared to other oncology patients. Furthermore, evidence confirms that hereditary RB survivors are at a higher risk for SMNs than nonhereditary RB survivors. Over the decades, some studies have been performed to better understand this subject, evaluating the risk of the development of SMNs in RB patients. Furthermore, this risk seems to increase with the use of ionizing radiation in some therapeutic approaches commonly used in the treatment of RB. This review aims to clarify the effect of ionizing radiation in RB patients and to understand the association between the risk of SMN incidence in patients that underwent radiation therapy, especially in hereditary RB individuals.

## 1. Introduction

Retinoblastoma (RB) is the most common primary intraocular tumor in children. Its worldwide estimated incidence ranges from 1 in 15,000 to 1 in 18,000 live births, showing no racial, ethnic, or gender predisposition [1,2]. Retinoblastoma’s survival rate can exceed 95% with early diagnosis and treatment. Thus, the survival rate in children diagnosed with RB in medically developed countries is significantly higher when compared to undeveloped countries due to the late diagnosis [3,4].

RB develops from the retinoblasts, precursors of retinal cells, and can be classified according to the disease’s laterality: unilateral, if only one of the eyes is affected, or bilateral (20–30% of cases) if both eyes are affected. Regarding its genetic classification, it is clinically classified as hereditary, representing about 45% of the diagnosed cases, or nonhereditary. Tumor development is associated with the retinoblastoma susceptibility gene (*RB1*), a recessive tumor suppressor gene involved in cell growth and development, and it is reported that it only occurs when both alleles of *RB1* are lost or undergo deletion, inactivation, or mutation [3,4,5]. In 1971, Knudson et al. [6] proposed the “two-hit” hypothesis, stating that RB is caused by two complementary chromosomal mutations. Thus, nonhereditary RB is associated with two somatic mutations, whereas hereditary RB is related to a germline mutation that would be present in all body cells, followed by a mutation in somatic retina cells. The offspring of hereditary RB patients are predisposed to the disease with a penetrance of 80% [3,4,5]. All cases of nonhereditary RB are associated with a unilateral tumor, whereas the hereditary form is mainly bilateral; however, in some cases, it can be unilateral. About 85% of unilateral RB result from somatic mutational events, and only 15% are related to hereditary RB [3,5]. However, the two mutational events that affect the *RB1* alleles seem to be insufficient for the formation of the malignancy, since a mutation in *RB1* first leads to retinoma, a benign precursor of RB [3,7]. In 2007, Corson and Gallie [8] stated that additional mutational events were required for the formation of a malignancy, supporting a “three-hit” hypothesis [9].

In most cases, the diagnosis of this malignancy is performed before an individual is 5 years old [2]. The most common symptomatic presentation in RB is leukocoria, an abnormal white reflex in the eye, followed by strabismus, representing 56% and 20% of all the observed signs, respectively [1,5]. Strabismus results from a loss of central vision when the tumor involves the macula, leading to ocular misalignment. Among the less common presenting signs are heterochromia, hyphema, glaucoma, and orbital cellulitis, which are all associated with more advanced stages of the disease at diagnosis [5]. An ocular examination under anesthesia, an indirect ophthalmoscopy fundoscopy to examine the entire retina, and a general physical examination are typically performed. Imaging studies—ultrasonography (US), magnetic resonance imaging (MRI), and, rarely, computed tomography (CT)—are also crucial to evaluate the presence of metastasis and the tumor’s size and to diagnose trilateral RB (bilateral retinoblastoma + pineoblastoma) [10,11].

The adoption of convenient therapy depends on the classification of the tumor stage by the International Classification of Retinoblastoma (ICRB). Group A comprises small tumors with a basal diameter or thickness equal to or less than 3 mm. Group B corresponds to tumors that are greater than 3 mm. The tumors can also be located 3 mm or less from the foveola, positioned ≤1.5 mm from the optic disc, or contain subretinal fluid ≤3 mm from the tumor margin. Group C comprises RB with contiguous seeds, i.e., RB with subretinal seeds 3 mm or less from the tumor, vitreous seeds ≤3 mm from tumor, or both subretinal and vitreous ≤3 mm from tumor. Group D involves RB with diffuse seeds, i.e., RB with subretinal seeds 3 mm or more from the tumor, vitreous seeds >3 mm from the tumor, or both subretinal and vitreous >3 mm from the tumor. Group E comprises extensive tumors (RB dominating more than 50% of the ocular globe or with neovascular glaucoma; opaque media from hemorrhages in the anterior chamber, vitreous, or subretinal space; or an invasion of the postlaminar optic nerve, choroid (>2 mm), sclera, orbit, or anterior chamber). This initial examination enables the clinicians to decide on the best treatment approach and avoid unnecessary side effects. Furthermore, the presence of extraocular clinical history and germline testing results, when available, are also considered during the choice of treatment [10]. Genetic testing is crucial for the early diagnosis in families with hereditary RB and often includes a complete sequencing of the coding region, an analysis of gene deletions, rearrangements, and methylation, and an RNA analysis. Since one assumes that patients with bilateral RB have a constitutional mutation, mutations are identified using a direct examination of the DNA from a blood sample. On the other hand, in unilateral RB patients, a blood sample and a tumor specimen, when enucleation is performed, are collected. Once the mutations are identified in the tumor, the same mutations are examined in the blood sample. If a mutation is identified in the blood, one assumes that it is a germline mutation. Once the germline mutation is confirmed, all patients’ siblings or offspring must be tested [5].

Retinoblastoma may lead to death when diagnosed and treated late, but only when an extraocular dissemination is noted. Regarding the treatment options for RB, enucleation is a nonconservative approach in which the eye globe is sacrificed. This is strongly recommended when there is an anterior segment invasion, neovascular glaucoma, intraocular hemorrhage, orbital cellulitis, and no potential for useful vision. It is performed mainly in group E of RB, with a suspected invasion of the optic nerve or choroid, and tumors that fail the conservative treatments [3,10,12]. In addition to enucleation, the available therapeutic approaches used for RB are conservative: radiation therapy, chemotherapy, transpupillary thermotherapy, and cryotherapy. Because external beam radiation therapy (EBRT) comprises many side effects and an increased risk of secondary tumors, it is no longer the elected primary treatment for RB patients, except when extraocular involvement occurs. Brachytherapy in RB management, involving radioactive sources such as Ruthenium-106 and Iodine-125, is typically used as a secondary treatment, carrying many advantages, such as minimal damage to the peripheral healthy areas surrounding the tumor, minimal risk of second tumors, and a shorter treatment period. However, side effects include cataracts, vitreous hemorrhages, and radiation retinopathy [1,10]. The introduction of chemotherapy in the group of conservative therapeutic approaches for RB provided an improvement in globe salvage, tumor control, and survival. Currently, intravenous chemotherapy (IVC) is the recommended primary treatment for bilateral RB when there is a germline mutation, a family history of RB, or a suspected invasion of the optic nerve or choroid. Intra-arterial chemotherapy (IAC) is quite successful for globe salvaging in advanced tumors, consisting of first-line therapy for somatic mutations and unilateral RB. Intravitreal chemotherapy (IvitC) is normally used as a second therapy in the presence of recurrent diffuse vitreous seeds for globe salvage after the failure of primary therapies. Therapeutic approaches derived from IvitC are also used: precision intravitreal chemotherapy (p-IvitC) for localized vitreous seeding and intracameral chemotherapy (IcamC), in which the anterior chamber is reached to treat aqueous seeding. Focal therapies, including transpupillary thermotherapy (TTT) and cryotherapy, are often used in combination with IVC or IAC to locally control small tumors [10,11,12]. Some modern therapeutic approaches have recently emerged, such as proton radiation therapy. The main advantage of its use lies in the fact that proton beams have no exit dose since these beams do not leave the tumor after they reach it, and, consequently, a smaller volume outside of the tumor receives radiation compared with the photon EBRT [13,14].

## 2. *RB1* Gene and Its Role in the Cell Cycle Control

*RB1* was molecularly defined and cloned in 1993. Back in the late 80s and early 90s, RB and its associated tumor suppressor gene played an important role in the cancer genetics field. It became possible to understand the biological function of this gene and the role of the retinoblastoma protein (pRB) [15]. *RB1* is located on 13q14.2, i.e., in region 14.2 of the long arm of chromosome 13. It contains 27 coding exons, 26 introns, and a core promoter spread over 178,143 bp. pRB has 928 amino acids in its composition [16].

**Figure 1:**
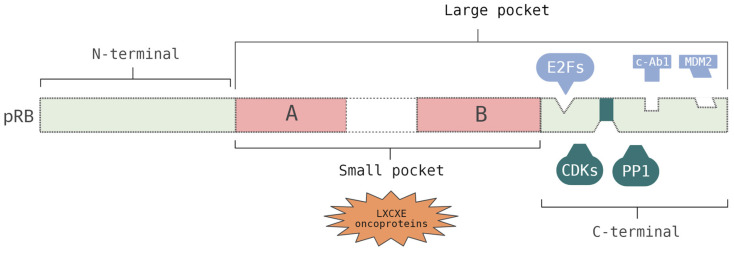
Representation of the protein encoded by *RB1*, the retinoblastoma protein, pRB. This protein comprises 928 amino acids. The small pocket (A/B domain separated by a spacer region) binds with the LXCXE motif of viral oncoproteins. The large pocket interacts with E2Fs, suppressing their transcription, and binds with the oncoproteins, c-Ab1 and MDM2. Furthermore, this region contains a short peptide region that is competitively bound by CDKs or PP1. Created with BioRender.com.

*RB1* belongs to a family of genes, known as the pocket family, which also includes the retinoblastoma-like 1 (*RBL1*) and the retinoblastoma-like 2 genes (*RBL2*) that code for the p107 and p130 proteins, respectively. The RB family of proteins (Figure 1) are divided into three main domains: the N-terminal (RbN), A/B pocket domain (RbAB), and C-terminal (RbC) [15,17]. The small pocket region comprises the A and B domains, which are separated by a spacer region, and interacts with the LXCXE motif that is found in viral oncoproteins, such as E1A and Tag. Each one of these domains (A and B) represents a single cyclin fold domain. The region comprising the small pocket and the C-terminal domain is denominated by a large pocket. This region can interact with E2F transcription factors (E2Fs), suppressing their transcription. Specifically, this interaction begins with the binding of the E2F transactivation domain to the cleft between the A and B domains, and a secondary interaction occurs in the C-terminal region. The C-terminal region is structurally undefined and possesses a docking site for the E2F1 transcription factor and a short peptide region that is competitively bound by cyclin/cyclin-dependent kinases (CDKs) or protein phosphatase 1 (PP1). The C-terminal fragment also binds to the oncoproteins, c-Abl and MDM2. Within the C-terminal, the deletion of exons 24 and 25 causes low-penetrance RB [7,17,18,19].

Mutations in *RB1* can be present in the germline or somatic cells. Only germline mutations can be inherited. Until 1999, a total of 368 mutations in this gene had been reported in the literature, of which about 62% represented single base substitution, 26% were small deletions, and 10% were small insertions [20]. In 2005, Valverde et al. [21] described a database with 500 distinct *RB1* mutations and more than 400 recurrences, of which 753 are germline mutations, 155 somatic mutations in RB tumors, and 24 somatic mutations in other types of tumors. About 42%, are nonsense mutations. However, this value diminishes to 18.6% if the recurrences are not considered. Therefore, a high recurrence is associated with nonsense mutations, while small insertions, deletions, and complex insertion–deletions show a low recurrence [21]. Inactivation of *RB1* is widely present in multiple types of malignant tumors, including prostate, lung, and breast cancers. Recent studies have shown that the pRB loss of function often present in retinoblastoma is mainly associated with nonsense mutations, frameshift mutations, and splice site mutations. Intronic, missense, and in-frame splice mutations can be associated with a partial loss of pRB function. Although *RB1* has been reported as a gene without a mutation hotspot, it has been stated that the RbAB pocket domain is rich in cancer-associated missense mutations and sites where a cytosine nucleotide is followed by a guanine nucleotide (CpG). These are potential mutation hotspots as nonsense mutations are often derived from these sites [16].

**Figure 2:**
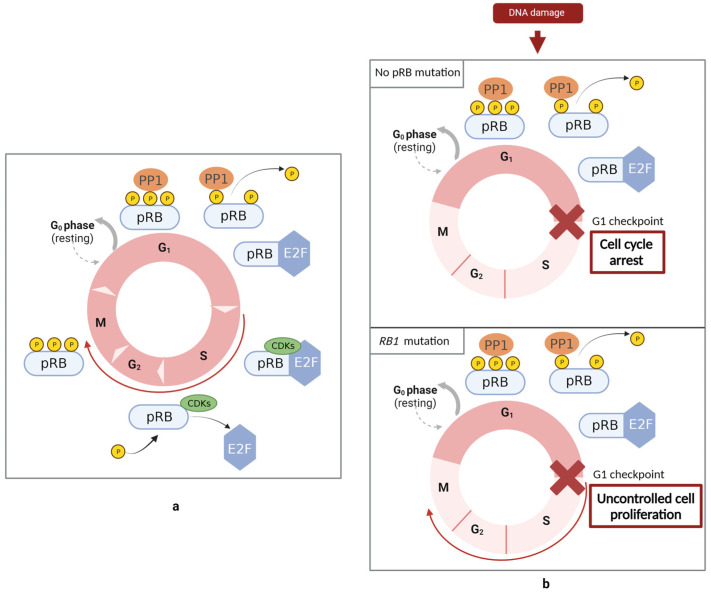
Role of pRB in the regulation of the cell cycle. (**a**) During the G1 phase of the cell cycle, pRB is dephosphorylated by PP1. The dephosphorylated form of this protein can form a complex with E2Fs, blocking its activity and, consequently, repressing transcription. E2Fs are responsible for the activation of the transcription of genes that encode the proteins required for the progression to the S phase, such as DNA polymerase, dihydrofolate reductase, and human cyclin-dependent kinase 1 (*CDK1*). In the final stages of G1 and continuing to the M phase, CDKs mediate pRB phosphorylation, leading to the release of E2F and, consequently, to the expression of genes that play an important role in cell division. Later in the cell cycle, the dephosphorylation of pRB by PP1 re-establishes pRB in the unphosphorylated form. (**b**) pRB is a negative regulator of the cell cycle. In response to DNA damage, CDKs are inhibited by the activation of checkpoints. pRB remains in the dephosphorylated form and bonded to E2F. Under these conditions, the cell is prevented from transitioning to the G1 checkpoint. The presence of a pRB mutation promotes uncontrolled cell cycle progression and, consequently, tumorigenesis. Created with BioRender.com.

The RB protein is a negative regulator of the cell cycle (Figure 2) since the loss of its function promotes uncontrolled cell cycle progression and, consequently, tumorigenesis. The function of this protein is regulated by phosphorylation reactions. Phosphorylation can occur at various sites of pRB, mainly in the spacer region between the A and B domains, in the interface between the RbN and pocket domain (RbIDL), and in the C-terminal region. During the G1 phase of the cell cycle, pRB is dephosphorylated. The dephosphorylated form of this protein can form a complex with E2Fs, blocking its activity and, consequently, repressing transcription. E2Fs are responsible for the activation of the transcription of genes that encode the proteins required for the progression to the S phase, such as DNA polymerase, dihydrofolate reductase, and human cyclin-dependent kinase 1 (*CDK1*). In the final stages of the G1 and continuing to the M phase, CDKs mediate pRB phosphorylation, leading to the release of E2F and, consequently, to the expression of genes that play an important role in cell division. Later in the cell cycle, the dephosphorylation of pRB by PP1 re-establishes pRB in the unphosphorylated form [20,22,23,24]. CDKs are a group of serine/threonine kinases that, in association with cyclin, regulate pRB phosphorylation. In response to DNA damage, these cyclin/CDK complexes are inhibited by the activation of checkpoints. Therefore, through the inhibition of the cyclin/CDK complex, pRB remains in the dephosphorylated form and bonded to E2F. As E2Fs are responsible for encoding proteins required for the progression to the S phase, under these conditions, the cell is prevented from transitioning to the G1 checkpoint, i.e., blocking the transition from the G1 phase to the S phase [25].

Although the mechanism of interaction between pRB and E2Fs is the most studied, evidence shows that it is not the only one involved in cell cycle control. The ability of pRB to suppress the target gene transcription through the recruitment of transcriptional corepressors and/or chromatin remodeling protein factors also prevents cell cycle progression. These repressors and protein factors are LXCXE proteins, since all of them can bind to the LXCXE motif of pRB, and include histone deacetylase (HDAC), replication factor C, the ATPase subunit of the SWI/SNF (SWItch/Sucrose Nonfermentable) complex with BRAHMA (Brm) and Brahma-related gene 1 (BRG1) proteins, DNA methyltransferase DNMT1, and heterochromatin protein HP1. Furthermore, pRB can physically interact with many nuclear proteins, such as histone methyltransferase Suv39h1, histone demethylase LSD1, and histone demethylase RBP2 (KDM5A) [24].

## 3. Ionizing Radiation

Radiation refers to the emission or transmission of energy in the form of particles or electromagnetic waves to a given material [26]. Considering its ability or inability to ionize matter, radiation can be classified as ionizing or nonionizing, respectively. Nonionizing radiation comprises radio waves, microwaves, infrared, visible, and ultraviolet light. This type of radiation has wavelengths equal to or greater than 10^−8^ m and energies lower than 12 eV. As the nomenclature indicates, ionizing radiation (IR) is able to ionize matter, i.e., remove electrons from atoms or molecules due to its high energy. This category of radiation can take the form of photons, such as X-rays and γ-rays, or particles, such as α particles, β particles, neutrons, and protons, among others. In clinical terms, IR is used for both diagnostic and treatment purposes [26,27,28]. In terms of diagnostic practices, exposure to IR comes mainly from X-rays in diagnostic radiology and from γ-rays from radioisotopes in nuclear medicine [27].

When passing through matter, IR undergoes a process of energy loss due to energy deposition and the interactions that occur. LET, linear energy transfer, refers to the density of energy deposition in a material, representing the average energy deposited per unit of length of the path of radiation, and it depends on the type of radiation, its energy, and the material’s density. Particulate radiation, such as protons, neutrons, and α particles, are classified as high-LET radiations, whereas X-rays, γ-rays, and β particles are low-LET. High-LET radiation can deposit the majority of its energy in the tumor’s area, allowing the surrounding tissues to be less affected [29,30,31,32]. The biological effect of the radiation is assessed using a parameter denominated by relative biological effectiveness (RBE), which depends on LET. In general, an increase in the radiation LET leads to an increase in its RBE. However, this fact only occurs up to a specific maximum LET level since, from then on, an increase in the value of LET will not contribute to the increase in the biological effect [31,32,33].

Despite its use in the medical context and its importance for the diagnosis and treatment of numerous diseases, IR can present adverse effects when their exposure is not properly controlled. According to the linear quadratic model, radiation effects may be classified as deterministic, characterized by an almost immediate impact and a specific threshold dose below which no clinical changes are observed; or stochastic, related to chronic effects with a long-term impact. The intensity of a deterministic effect is directly proportional to the dose deposited, and the effect is only observed at doses higher than the threshold value. In contrast, stochastic effects are independent of a threshold dose, meaning that low doses can also induce changes. Carcinogenesis, genetic mutations, and chromosome aberrations are examples of stochastic effects [26,33,34]. Furthermore, the biological effects of radiation can be direct or indirect. The direct action of IR is mainly associated with DNA, but it can also affect other macromolecules crucial for the survival of the cell. The direct effects of IR include structural changes, cell damages, and even cell death. Moreover, it is normally associated with higher doses and high-LET radiations (e.g., α particles and neutrons). On the other hand, the indirect effects of IR are mainly associated with water radiolysis. In this process, water molecules are hit by ionizing radiation, leading to the production of free radicals, very reactive species that are susceptible to reacting with DNA molecules and, potentially, can cause DNA damage. The indirect action of IR, mainly associated with low-LET radiation, triggers most of the cellular damage since water makes up about 70% of the cell’s constitution [29].

The linear quadratic model also states that low doses of ionizing radiation cause mainly single-strand breaks (SSBs), whereby only one DNA strand is damaged, while high doses would normally cause double-strand breaks (DSBs) that are more difficult to repair. Only a considerable dose of IR can incite irreparable cellular damage [34]. Effectively, the DNA damage caused by ionizing radiation includes base damage, strand breaks (SSBs or DSBs), sugar damage, DNA crosslinking, and clustered damaged sites. Base damages and SSBs present a low biological impact, being easily reparable with the support of the intact complementary strand. In contrast, DSBs are more frequent and difficult to repair and can easily lead to major damage, including carcinogenesis or cell death. Most DSBs can be correctly repaired essentially through nonhomologous end joining (NHEJ), which is dominant in the G1 and S phases of the cell cycle but present throughout the cell cycle, and homologous recombination (HR), operating only in the late S or G2 phases. During the HR repair process, after the recognition of the DSB, DNA strands near the DSB are resected, and homologous DNA strands serve as templates to repair the damaged DNA. The 5′ ends of the DSB are excised to form a 3′ end single-stranded DNA (ssDNA) using specific nucleases, which will pair with the homologous double-stranded DNA forming a D-loop structure that later will be dissociated. Many repair proteins play important roles in HR, such as Rad51, Rad52, RPA, BRCA2, PALB2, and CtIP. In NHEJ, the dominant repair process for DSBs, damaged or mismatched nucleotides are removed after the rapid and easy connection of the two broken DNA ends. The DNA structure is reestablished after the strands bind following the recruitment of proteins, like DNA-dependent protein kinase (DNA-PKCs), and enzymes, such as Artemis, polynucleotide kinase phosphatase (PNKP), human apurinic endonuclease (APE1), and tyrosyl-DNA phosphodiesterase 1 (Tdp1). NHEJ can incite small mutations due to the loss of genetic material caused by end-processing and the binding steps [31,35,36].

## 4. Risk of Second Tumor Incidence in Survivors of Retinoblastoma Treated with Radiation Therapy

The main cause of death among hereditary RB survivors remains to be the occurrence of subsequent malignant neoplasms (SMNs). SMNs are new tumors that develop after the incidence of a primary tumor. While some authors defend that an SMN is histologically independent from the first primary tumor, others state that trilateral RB has to be reported as an SMN. As already mentioned, all cells of hereditary RB patients carry a germline mutation in one allele of *RB1*, a tumor suppressor gene. On the other hand, nonhereditary RB is associated with mutations that occur only in retina cells. Therefore, survivors of hereditary RB seem to present a higher risk of developing SMNs compared to the general population as well as nonhereditary RB survivors [37]. Thus, this section aims to analyze some studies (Table 1) that have been performed to compare the incidence of SMNs between hereditary and nonhereditary RB and also to evaluate the risk of developing SMNs after treatment with RT in RB patients [37].

Mohney et al. (1998) [38] developed a retrospective cohort study for a 50-year period to correlate the incidence of second nonocular tumors in 180 survivors of RB with the RT protocol these patients underwent. This study was based on the assessment of clinical data records to gather information from all the children involved, such as gender, laterality of the disease (unilateral or bilateral), family history, age, and the therapeutic approach used to manage the tumor. About 46% (82 children) had hereditary RB. In 15 of these 82 patients, 16 nonocular tumors were identified, of which six were soft tissue tumors, four bone tumors, three melanomas, and three carcinomas (one of the breast, one pancreas, and one thyroid). The data showed that these patients received an average dose of 4605 cGy (=46.05 Gy) during treatments. The overall results showed that there were 82 patients with hereditary RB, of which 14 patients have undergone RT, and second tumors were only noticed inside the irradiation field in 4 patients. Thus, this study’s results showed no relevant association between the development of SMNs in survivors of hereditary RB and the previous exposure to radiation.

In 2005, a large study involving 1601 1-year survivors of RB was carried out by Kleinerman et al. [39], whose purpose was to evaluate their long-term predisposition to SMNs when treated with RT. These patients were diagnosed from 1914 to 1984 and their follow-up period began 1 year after diagnosis and extended until 2000 or until their death. This study identified 963 survivors with hereditary RB, of which 47 are unilateral and 916 bilateral. The remaining patients, about 40%, were associated with nonhereditary unilateral RB. Regarding the type of treatment that the irradiated patients (849) underwent, 90% were associated with EBRT, 1% with brachytherapy, and the remaining 9% had a combination of both. The average dose was 48 Gy, ranging from 15 to 115 Gy. The subsequent cancer risk was determined using the standardized incidence ratio (SIR), which was calculated as the ratio between the number of observed tumors and the expected number of tumors. According to the results of this study, hereditary RB patients presented a significantly higher risk (SIR = 19). However, the same did not apply to the nonhereditary form of the disease (SIR = 1.2). The results showed that the hereditary form of the disease presents an increased risk of developing cancers of the nasal cavity, eye, orbit, brain, and lung. Furthermore, regarding the effect of RT, it most likely contributed to the observed increased risk for brain, nasal cavity, eye, and orbit cancers. However, the increased risk associated with melanoma and corpus uteri, colon, and breast cancers is probably dissociated from the radiation, since the effect was similar in both irradiated and nonirradiated patients.

Later, in 2008, Marees et al. [40] performed a 40-year follow-up on 668 Dutch survivors of RB, aiming to collect conclusions about the long-term incidence of subsequent epithelial cancer. About 70% of the hereditary RB patients were treated with RT compared to only 8% of the nonhereditary. There were 62 detected SMNs, associated with an SIR of 20.4, among hereditary RB patients. On the other hand, the group of nonhereditary RB patients comprised 12 SMNs with an SIR of 1.86. When compared to the SIRs related to all cancer sites, the results showed a statistically increased risk of soft tissue sarcomas in nonhereditary RB patients; however, it is not related to radiation. About 89% of SMNs in hereditary RB patients were found in patients treated with radiation therapy, and 40% were in-field soft tissue sarcomas, cancer of the bone, or melanoma. Among hereditary RB patients, this study reported an elevated risk of developing not only bone cancer, soft tissue sarcoma, and melanoma, consistent with other studies performed, but also epithelial cancers, including bladder, lung, and breast cancers. These results are probably associated with the presence of both an *RB1* germline mutation and radiation exposure.

MacCarthy et al. (2013) [41] conducted a follow-up study involving 1927 RB patients (806 hereditary and 1121 nonhereditary), diagnosed in Britain between 1951 and 2004, aiming to investigate the incidence of SMNs. The authors identified 169 SMNs in 152 patients, in which 146 were found in heritable cases and 23 in nonheritable cases. This fact reports an increased incidence of SMNs in the hereditary RB compared to the nonhereditary. Considering the number of SMNs developed, 11 patients developed two SMNs, and 3 patients developed three. Thus, it becomes extremely important to follow-up with survivors and respective family members in these cases. Furthermore, it is relevant to mention that heritable RB survivors present a higher risk of leiomyosarcoma incidence. Regarding the effect of radiation, among the hereditary RB patients, there were 45 detected SMNs in the head and neck region (region of potential exposure to radiation) and 70 outside this region (outside the irradiated field). In contrast, among nonhereditary patients, 6 SMNs were identified in the head and neck region and 14 outside. This study’s results showed no relevant association between radiation exposure and the development of second tumors.

A long-term follow-up study from 1943 to 2013, aiming to examine the incidence and mortality of RB patients with SMNs in a cohort of 323 Danish RB patients (133 hereditary and 190 nonhereditary), was carried out by Gregersen et al. (2020) [42]. Among patients with hereditary RB, a total of 25 SMNs were observed, namely 14 sarcomas, six melanomas, three carcinomas (two breast and one ovarian cancer), and two central nervous system (CNS) tumors. In the group of nonhereditary RB, only 13 cases were detected: five carcinomas (two lung, one breast, one cervical cancer, and one thyroid cancer), four sarcomas, three melanomas, and one CNS tumor. These facts led to the presumption that the risk for SMN incidence in hereditary RB is significantly higher compared to the nonhereditary RB group. Patients with hereditary RB were treated with EBRT (group 1), brachytherapy (group 2), or without radiation therapy (group 3). The therapeutic approaches in group 3 included enucleation, enucleation + chemotherapy, chemotherapy alone, or chemotherapy + laser transpupillary thermotherapy. Among a total of 76 hereditary RB patients treated with EBRT, five of the eight sarcomas were observed inside the irradiated field. On the other hand, among a total of 57 hereditary patients treated without EBRT, none of the six observed sarcomas were localized inside the field of irradiation. However, no overall increased risk was detected for SMN incidence in hereditary RB patients treated with external radiation therapy. Furthermore, the cumulative mortality rate from SMNs was significantly higher for hereditary RB patients.

More recently, in 2021, Schonfeld et al. [43] carried out a long-term study involving 2052 American survivors of RB (1128 hereditary and 924 nonhereditary). The main purpose was to clarify whether risks for epithelial cancers are increased, since previous studies reported a statistically significant increased risk for some epithelial tumors. However, the sample sizes in those studies were small. Additionally, their investigation was focused on quantifying the risks for the incidence of multiple SMNs in hereditary RB survivors. An increased incidence of SMNs in the breast, CNS, nasal cavity, and pineoblastoma compared to other types of epithelial cancer among hereditary RB survivors was verified by Schonfeld et al. However, unlike Marees et al., the increased incidence was not observed for kidney, bladder, uterus, pancreas, and lung malignancies.

In 2021, Zhao et al. [44] performed a cohort study involving 62 hereditary RB patients and nonhereditary RB patients previously treated with RT (56%) or chemotherapy (27%). The main goal of this study was to establish an association between radiation or chemotherapy exposure and SMN incidence in these patients. Regarding the number of a second primary cancer, 41 patients developed one SMN, 11 developed two SMNs, and 5 of them had three SMNs. Among other types of SMNs, 51 sarcomas, 11 skin cancers, and 7 breast cancers were reported. In general, bilateral RB is also associated with both a higher number and a wider spectrum of SMNs. However, this study showed no association between treatment with RT or chemotherapy and the risk of SMN incidence, probably due to the small number of patients involved.

Sethi et al. (2013) [14] carried out a retrospective study reporting on a cohort of 86 patients, of which 55 were treated with proton therapy and 31 with photon RT. Among the 55 patients in the proton cohort, 84% of them had hereditary RB compared to 61% in the photon cohort. In the proton cohort, 56% of the patients involved were also treated with chemotherapy, whereas only 16% of the photon cohort received chemotherapy. Among the 55 patients treated with proton therapy, only 1 patient (hereditary RB) developed an SMN outside the field of irradiation, an osteosarcoma. In the photon cohort, four SMNs (only one in a hereditary RB patient) were identified, all of them inside the field of irradiation: three sarcomas (orbital, maxillary, and temporal bone) and one glioblastoma multiforme. Despite the short average follow-up time of these patients, these facts testify that, compared to photon therapy, the risk of SMNs associated with radiation is significantly lower when patients are treated with proton therapy.

Mouw et al. (2014) [45] studied a cohort of 49 RB patients that were also treated with proton radiation therapy, with a median follow-up of 8 years. Bilateral disease was detected in 41 patients. The treatment with protons was performed in 60 eyes/tumors, of which 27 were classified as early-stage (ICRB groups A-B), 31 were advanced-stage (ICRB groups C-D), and 2 were not able to be classified. About 49.51% of the patients involved were also treated with chemotherapy. Focal techniques, such as laser photocoagulation and cryotherapy, were used in the treatment of 43 tumors. The median radiation dose was 44 Gy (effective dose). Twelve of the sixty irradiated tumors, mainly advanced tumors, developed ocular complications due to radiation treatment, such as cataracts, radiation retinopathy, glaucoma, neovascularization, and others. This study’s results revealed only one SMN, an osteosarcoma of the femur outside the irradiation field in a hereditary RB patient.

The reviewed studies confirm that hereditary RB patients are at a higher risk for the development of subsequent malignant neoplasms than nonhereditary RB, since a higher number of SMNs is noted in the hereditary groups of patients. In fact, this is especially notorious in studies with larger sample sizes, e.g., Kleinerman et al. (2005) [39], MacCarthy et al. (2013) [41], and Schonfeld et al. (2021) [43]. Thus, there seems to be a trend toward consensus on this matter. The fact that, in nonhereditary RB, the mutational events in *RB1* occur only in somatic cells of the retina, seems to justify the lower number of SMNs in these cases. The most common types of second primary tumors observed are soft tissue sarcomas, bone tumors, and melanomas. In some studies, a high incidence of leiomyosarcoma, brain/CNS tumors, breast cancer, and nasal cavity tumors is also reported (Table 1).

Furthermore, regarding the possible association between the radiation exposure of RB patients and the development of subsequent malignant neoplasms, we observed an increasing number of SMNs in irradiated patients. However, Kleinerman et al. (2005) [39], MacCarthy et al. (2013) [41], Gregersen et al. (2020) [42], Schonfeld et al. (2021) [43], and Zhao et al. (2021) [44] were clear about the absence of a significant association between ionizing radiation exposure and the development of SMNs, even though they reported second primary tumors inside the field of irradiation and an increasing number of SMNs after radiation therapy. In addition, it was also clear that there is a higher number and a wider spectrum of SMNs in hereditary RB cases. This is probably explained by both an *RB1* germline mutation and radiation exposure in these patients.

Aiming to clarify the risk of second primary tumors in patients treated with proton therapy, both Sethi et al. (2013) [14] and Mouw et al. (2014) [45] reported no in-field of irradiation SMNs and only one SMN outside the irradiation field. Sethi et al. developed a more complete study that compared the development of SMNs in a cohort of patients that underwent photon therapy vs. patients treated with proton therapy. In the photon cohort, four in-field SMNs were observed. Thus, even though it brings some ocular complications, proton therapy seems to be a safer approach for the treatment of RB.

## 5. Conclusions

This review has allowed for the confirmation of a higher predisposition for SMNs in hereditary RB patients compared to nonhereditary RB patients. Even though the increase of SMNs in irradiated patients is clear, especially when that exposure is associated with photon therapy, there is no significant association between exposure to IR and the incidence of SMNs in survivors of RB. Thus, these conclusions seem to justify why radiation therapy tends to be avoided as the primary treatment approach to RB. However, knowing in advance that RBs tumors are highly responsive to radiation therapy and that, in some cases, especially when extraocular involvement occurs, EBRT is utilized, it is extremely important to carry out more studies on this matter. Future studies should expand the sample size, increase the follow-up time, and be complemented with radiobiological studies.

## Figures and Tables

**Table 1:** Studies carried out to examine the risk of SMN incidence in survivors of RB when treated with radiation therapy.

Authors	Sample Size	Treatment (# Patients)	Type of SMNs	Treatment Related to SMNs (# Patients) ^a^	SMNs Location (# SMNs) ^b^
Mohney et al. (1998) [38]	180 patients: - 82 hereditary - 98 nonhereditary	Hereditary: RT (60); ChT (15); Unknown (7) Nonhereditary: Not disclosed	Hereditary: 16 (6 soft tissue tumors, 4 bone tumors, 3 melanomas, 3 carcinomas—1 breast, 1 pancreas, and 1 thyroid) Nonhereditary: 3 (1 benign soft tissue tumor and 2 carcinomas—cervical and breast)	Hereditary: EBRT (6); EBRT + CT (4); RA (3); EBRT + BT + RA (1); ChT (1); Unknown (1) Nonhereditary: RA (1); Unknown (2)	Hereditary: In-field of irradiation (4); outside field of irradiation (10) Nonhereditary: In-field of irradiation (1)
Kleinerman et al. (2005) [39]	1601 patients: - 963 hereditary - 638 nonhereditary	Hereditary: Surgery (94); ChT (16); RT (466); RT + ChT (383); Unknown (4) Nonhereditary: Surgery (480); ChT (40); RT (67); RT + ChT (47); Unknown (4)	Hereditary: 260 (75 bone, 34 connective and soft tissue, 32 nasal cavities, 29 cutaneous melanomas, 17 eye and orbit, 10 brain, 10 female breast…) Nonhereditary: 17 (7 female breast, 2 brain, 2 thyroid, 1 Hodgkin’s lymphoma, 1 leukemia…)	Hereditary: RT (241); No RT (19) Nonhereditary: Not disclosed	Not disclosed
Marees et al. (2008) [40]	668 patients: - 298 hereditary - 370 nonhereditary	Hereditary: Surgery (70); ChT (16): RT (152); RT + ChT (58); Unknown (2) Nonhereditary: Surgery (322); ChT (8): RT (22); RT + ChT (8); Unknown (10)	Hereditary: 62 (20 soft tissue, 16 bone tumors, 13 melanomas, 11 epithelial—4 bladder, 3 lung, 2 breast, and 2 non-Hodgkin lymphomas) Nonhereditary: 12 (5 solid cancers, 3 soft tissue, 2 lung, and 2 leukemia)	Hereditary: EBRT (38); EBRT + ChT (17); Surgery (3); Laser coagulation (3); Unknown (1) Nonhereditary: Surgery (12)	Hereditary: In-field of irradiation (22); outside field of irradiation (33) Nonhereditary: In-field of irradiation (0); outside field of irradiation (0)
MacCarthy et al. (2013) [41]	1927 patients: - 806 hereditary - 1121 nonhereditary	Not disclosed	Hereditary: 146 (33 leiomyosarcoma, 33 osteosarcomas, 14 melanomas, 15 brain/CNS, 9 female breast, 9 meningiomas, 8 bladder…) ^c^ Nonhereditary: 23 (3 osteosarcomas, 4 brain/CNS, 3 meningioma, 2 female breast, 2 melanomas…) ^c^	Not disclosed	Hereditary: In-field of irradiation (45); outside field of irradiation (70) Nonhereditary: In-field of irradiation (6); outside field of irradiation (14)
Gregersen et al. (2020) [42]	323 patients - 133 hereditary - 190 nonhereditary	Hereditary: RT (31); RT + ChT (6); RT + ChT + enucleation (13); RT + enucleation (93); ChT (2); ChT + enucleation (7); enucleation (171) Nonhereditary: RT (25); RT + ChT (6); RT + ChT + enucleation (11); RT + enucleation (75); ChT (2); ChT + enucleation (2); enucleation (12)	Hereditary: 25 (14 sarcomas, 6 melanomas, 3 carcinomas—2 breast and 1 ovarian cancer—and 2 CNS) Nonhereditary: 13 (5 carcinomas—2 lung, 1 breast, 1 cervical cancer, and 1 thyroid cancer—4 sarcomas, 3 melanomas, and 1 CNS)	Hereditary: EBRT (13); No EBRT (12) Nonhereditary: EBRT (0); No EBRT (13)	Hereditary: In-field of irradiation (6); outside field of irradiation (7) Nonhereditary: In-field of irradiation (0); outside field of irradiation (0)
Schonfeld et al. (2021) [43]	2052 patients: - 1128 hereditary - 924 nonhereditary	Hereditary: RT (550); RT + ChT (435); ChT (39); Surgery (90); Unknown (14) Nonhereditary: RT (101); RT + ChT (86); ChT (61); Surgery (636); Unknown (40)	Hereditary: 265 (89 soft tissue sarcomas, 80 bone tumors, 28 melanomas, 12 breast, 11 nasal cavity, 8 pineoblastoma, 6 CNS…) ^d^ Nonhereditary: 27 (8 breast, 3 melanomas, 3 gastrointestinal, 2 thyroid, 2 lung…) ^d^	Not disclosed	Not disclosed
Zhao et al. (2021) [44]	62 patients - 40 hereditary - 17 nonhereditary - 5 unknown	RT (35); Chemotherapy (17); Surgery (16); Unknown (5) ^e^	Hereditary: 40 (37 sarcomas, 4 breast cancers, 3 adenocarcinoma, 2 meningiomas, 2 thyroid carcinoma...) Nonhereditary: 17 (11 sarcomas, 4 carcinomas...)	Hereditary: RT (27); No RT (10) Nonhereditary: RT (7); No RT (10)	Not disclosed
Sethi et al. (2013) [14]	86 patients	Proton therapy (55) Photon therapy (31)	Proton therapy: 1 (osteosarcoma) Photon therapy: 4 (3 sarcomas—orbital, maxillary, temporal bone—and 1 glioblastoma multiforme)		Proton therapy: In-field of irradiation (0); outside field of irradiation (1) Photon therapy: In-field of irradiation (4); outside field of irradiation (0)
Mouw et al. (2014) [45]	60 tumors ^f^	PRT (60) ^g^	Hereditary: 1 (osteosarcoma of the femur) Nonhereditary: 0		Hereditary: In-field of irradiation (0); outside field of irradiation (1) Nonhereditary: In-field of irradiation (0); outside field of irradiation (0)

^a^ Treatment approaches in patients who developed SMNs; ^b^ Location of SMNs in the irradiated group of patients. This classification between in-field and outside the field of radiation is based on the region where the SMNs develop (within the head and neck region is considered in-field of irradiation); ^c^ Among the hereditary group of patients, 146 SMNs were detected in 112 patients, whereas in the nonhereditary group of patients, 23 SMNs were detected in 20 patients; ^d^ Among the hereditary group of patients, 265 SMNs were detected in 239 patients, whereas in the nonhereditary group of patients, 27 SMNs were detected in 25 patients; ^e^ Data not disclosed regarding therapeutic approaches by hereditary/nonhereditary group; ^f^ Data show 60 tumors in 49 RB patients; ^g^ Among this group of patients treated with PRT, 25 patients had also received chemotherapy, and 43 tumors were treated with cryotherapy/laser; #: number; RT: radiation therapy (not specified); EBRT: external beam radiation therapy; BT: brachytherapy; RA: radium implants; ChT: chemotherapy; PRT: proton radiation therapy.

## Data Availability

No new data were created. The data retrieved from bibliography are within the article.
